# Cell fate acquisition and reprogramming by the proneural transcription factor ASCL1

**DOI:** 10.1098/rsob.250018

**Published:** 2025-06-18

**Authors:** Jethro Lundie-Brown, Francesca Puletti, Anna Philpott, Roberta Azzarelli

**Affiliations:** ^1^ Cambridge Stem Cell Institute, University of Cambridge, Cambridge, UK; ^2^ UK Dementia Research Institute, University College London, London, UK; ^3^ Department of Oncology, University of Cambridge, Cambridge, UK; ^4^ Department of Pharmacology, UCL School of Pharmacy, University College London, London, UK

**Keywords:** neurogenesis, fate reprogramming, chromatin remodelling, proneural transcription factors, ASCL1

## Abstract

ASCL1 is a key member of the proneural basic helix-loop-helix (bHLH) transcription factor (TF) family and it plays diverse roles in nervous system development and maintenance. ASCL1 is also one of the most studied bHLH TFs in the field of somatic cell reprogramming, as it can reconfigure the chromatin of the cell of origin to impose a neuronal identity. However, the ability of ASCL1 to drive neuronal fate does not come without exceptions, as there are cell types that are refractory to ASCL1-mediated reprogramming, and there are developmental contexts where ASCL1 does not drive neurogenesis but supports the generation of other lineages. ASCL1 has also emerged as an important player in cancers like neuroblastoma and glioblastoma, underscoring the clinical need for a robust understanding of how ASCL1 controls cell identity. In this review, we revisit the foundational studies that established ASCL1 as a critical regulator of neuronal differentiation and incorporate recent advances in our understanding of ASCL1 post-translational regulation and transcriptional control. By integrating these perspectives, this review provides a comprehensive overview of the diverse roles of ASCL1 in development, reprogramming and cancer, offering insights into its molecular functions and therapeutic potential.

**Figure:**
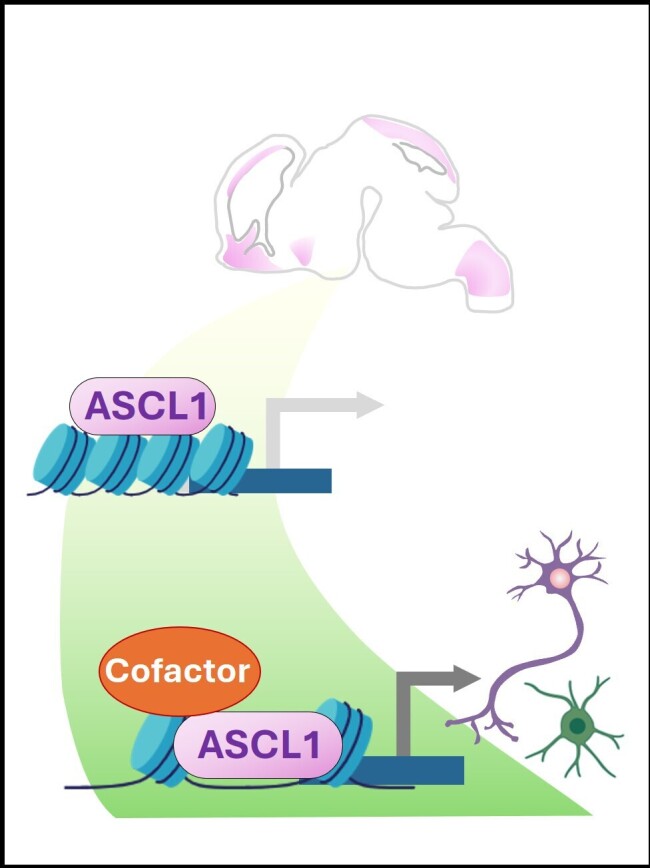

## 1. Introduction

Proneural transcription factors (TFs) make up a class of related proteins that regulate the expression of neural genes and drive neuronal differentiation [1,2]. They display a high level of structural homology, centred around the conserved basic helix-loop-helix (bHLH) region [3]. Despite this similarity, proneural TFs display a diverse range of functions in the development and maintenance of the central and peripheral nervous systems [4,5]. ASCL1 is one of the best characterized proneural bHLH TFs; it plays essential roles in the regulation of neural stem cell proliferation and differentiation, balancing lineage decisions and controlling the stem cell-like states of neurological cancers [4,6,7]. ASCL1 has also been identified as a key player in the reprogramming of somatic cells into neurons *in vivo* and *in vitro* [8–10]. In this context, it has been proposed to act as a pioneer TF, remodelling the chromatin landscape to increase competence for neuronal differentiation. Thus, ASCL1 plays multifaceted roles in development, fate reprogramming and cancer via mechanisms that go beyond activating transcription.

In this review, we provide a comprehensive overview of the varied functions of ASCL1 in these different contexts. We discuss recent works focusing on ASCL1’s control of gene transcription and chromatin remodelling and contextualize these advances in our understanding of the complex regulation of neuronal differentiation.

## 2. The multiple roles of ASCL1 in nervous system development

The mammalian *ASCL1* was named after the homologous gene originally identified in *Drosophila*, *achaete-scute*, which was implicated in sensory organ development, where it drives neuronal differentiation from a pool of equipotent ectodermal cells [11]. In mammals, however, ASCL1 is expressed in progenitor cells that already display some level of neuronal commitment and it functions as a downstream modulator of differentiation and subtype specification [12]. ASCL1 is predominantly recognized for its role in driving neuronal differentiation of the inhibitory GABAergic lineage during development [12–14]. However, recent research has revealed a far more nuanced and extensive role for ASCL1 than previously understood. Beyond its well-established function in specifying neuronal precursor cells throughout the central nervous system (CNS) [15], ASCL1 also directs the development of oligodendrocyte precursor cells in the spinal cord and cortex [11], as well as Müller glia in the retina [16,17]. Moreover, ASCL1 contributes to the maintenance of the stem cell pool in adult neurogenic niches, long after the developmental programmes have terminated [15]. Thus, understanding the molecular underpinning of ASCL1 context-dependent functions is key to develop reprogramming strategies to instruct specific cell identities.

### 2.1. ASCL1 in the peripheral nervous system

**Figure 1.**
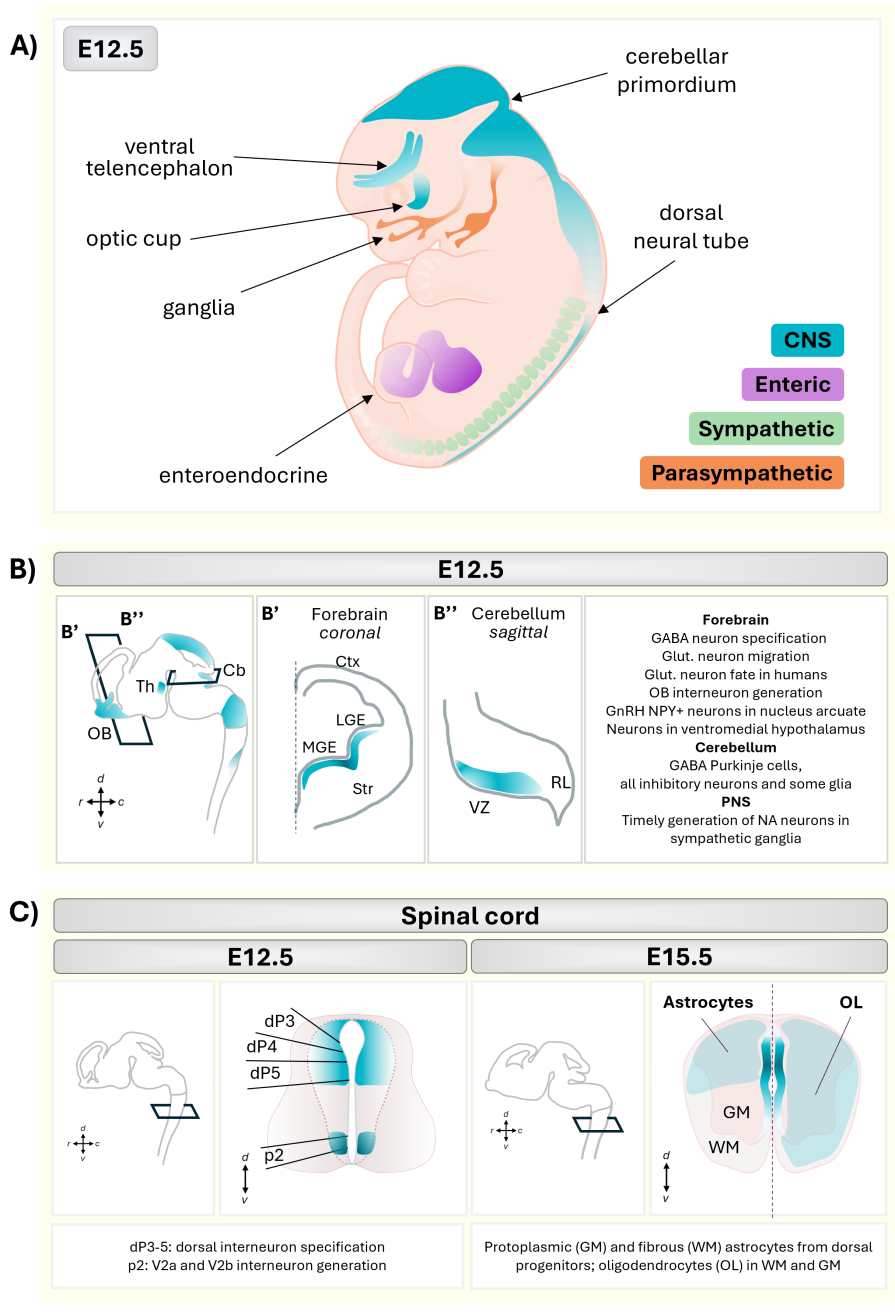
*Ascl1* expression and functions in the developing mouse embryo. (A) Schematic representation of mouse embryo at embryonic day (E) 12.5. *Ascl1* is expressed in the central nervous system (CNS, blue) and all the three major subdivisions of the peripheral nervous system (PNS): the enteric (purple), the sympathetic (green) and the parasympathetic (orange) nervous system. (B) Expression of *Ascl1* (blue) in the embryonic mouse brain at E12.5. (B’) Coronal section of the forebrain, showing *Ascl1* expression in the medial and lateral ganglionic eminences (MGE, LGE). (B’’) Sagittal section of the cerebellar primordium, showing *Ascl1* expression in the ventricular zone (VZ), but not in the adjacent rhombic lip (RL). Key cell types with *Ascl1* expression are listed by region (right)—telencephalon: GABAergic neuron specification [13,24–26], glutamatergic neuron migration [27,28], glutamatergic neuron fate in humans [29], olfactory bulb (OB) interneuron generation [30–33], GnRH+ NPY+ neurons in nucleus arcuate [34], neurons in ventromedial hypothalamus [35]; cerebellum: GABA Purkinje cells, all inhibitory neurons and some glia [36–40]; PNS: timely generation of NA neurons in sympathetic ganglia [21,41]. Abbreviations: OB, olfactory bulb; Th, thalamus; Cb, cerebellum Ctx, cortex; Str, striatum. (C) Expression of *Ascl1* (blue) in the spinal cord at E12.5 (left) and E15.5 (right). Dorsal domains (dP3, dP4, dP5) and the ventral domain p2 (highlighted in blue) contain Ascl1+ progenitors that give rise to sensory and motor neurons. From E15.5 onwards (right panels), *Ascl1-*expressing progenitors give rise to both astrocytes and oligodendrocytes (OL). Both cell types populate the white matter (WM) and the grey matter (GM) of the spinal cord, but astrocytes show a dorsal bias in their localization. dP3−5: dorsal interneuron specification [42], p2: V2a and V2b interneuron generation [43,44]; protoplasmic (GM) and fibrous (WM) astrocytes from dorsal progenitors, oligodendrocytes (OL) in WM and GM [45,46]. Elements created in BioRender (https://BioRender.com/44u8pk3).

ASCL1 is expressed in all three subdivisions of the mammalian peripheral nervous system (PNS): sympathetic [18], parasympathetic [19,20] and enteric [20–23] (figure 1A). Knockout of *Ascl1* in mice results in the near-total eradication of the olfactory system, the paracardiac ganglia and a class of early born neurons in the enteric nervous system [21,22]. In the sympathetic PNS, ASCL1 is expressed in sympatho-adrenal progenitors derived from the neural crest [12,22] and plays important functions in this lineage. *Ascl1* knockout embryos survive until birth, but die shortly after due to breathing and feeding defects [22]. Notably, these embryos successfully produce β-hydroxylase (Dbh)-positive noradrenergic neurons, albeit after a developmental delay [22,47,48]. Lineage tracing of neural crest (NC)-derived cells in *Ascl1−/−* mice revealed that NC-derived cells could still populate the sympathetic trunk and basal ganglia [49]. However, loss of *Ascl1* produces significantly smaller sympathetic ganglia in these mice due to impaired proliferation [49]. Taken together, these findings indicate that while ASCL1 is not absolutely essential for the generation of noradrenergic neurons, it plays a critical role in the timely specification and expansion of these cells. The developmental delay and incomplete population of the ganglia suggests that ASCL1 is compensated for by some other proneural factors. Indeed, ectopic expression of the related proneural bHLH TF *Neurog2* can partially rescue loss of *Ascl1*, by generating noradrenergic neurons in the sympathetic ganglia. Similarly, ASCL1 can partially rescue Neurog2 knockout phenotype in the peripheral and central nervous systems, indicating that there is some overlap in the function of bHLH TFs in these contexts [50].

### 2.2. ASCL1 in the central nervous system

ASCL1 controls cell fate throughout the development of the CNS, including in the forebrain [13,24,29,50–53], retina [54–57], olfactory bulb [30,31], spinal cord [42,58], hypothalamus [35] as well as in the cerebellum [36] (figure 1).

A summary of ASCL1 functions in these different regions is shown in figure 1; here we will mainly discuss its role in forebrain development. In this context, ASCL1 has been characterized as the main driver of GABAergic interneuron identity, by contrast to alternative proneural factors, like Neurog2, which specify the glutamatergic neurons [24]. However, there is increasing evidence, from works in mice and also in humans, that ASCL1 plays a role in both excitatory and inhibitory cell types [27,29,59–63].

ASCL1+ progenitors in the medial and lateral ganglionic eminences of the ventral telencephalon generate GABAergic interneurons that populate the striatum or migrate towards the cerebral cortex (figure 1B) [4,7,13,24–26]. GABAergic cell specification is initiated by ASCL1 through the activation of a transcriptional cascade composed of *Dlx* genes 1, 2, 5 and 6, and *Lhx6* [64–68]. By contrast, in dorsal cortical progenitors, *Ascl1* is expressed at low levels, partially overlapping with *Neurog2* [69], and its role is less precisely defined. When *Ascl1* is expressed in place of *Neurog2* [50] or overexpressed via *in utero* electroporation [69] in the embryonic cortex, it can respecify the identity of the neurons generated from cortical neural progenitors (from glutamatergic to GABAergic neurons). This ability to respecify progenitor subtype identity is confined only to early stages of corticogenesis [69], while in mid-corticogenesis ASCL1 promotes glutamatergic progenitor cell maturation through its effects on cytoskeletal remodelling and neuronal migration [28,69]. Clearly the effect of ASCL1 overexpression is dependent on the initial state of the target cells and temporal changes in the ability to respecify fate identity may reflect some progressive lineage restriction or changes in competence for differentiation.

It has also been shown that ASCL1 plays a role in the correct layering of the cortical glutamatergic neurons [60]. Cortical glutamatergic neurons populate six distinct layers of the cortex in an inside-out pattern of migration, with lower layer neurons being born early in development and upper-layer neurons being born later and migrating above the previously settled neurons. ASCL1 is involved in regulating the temporal transitions between laminar fates, which ensure proper development of the cortical layers, through a repressive network of interacting genes (*Tbr1*, *Fezf2*, *Satb2*, *Ctip2*) in postmitotic neurons [60]. When ASCL1 expression is altered, the temporal window of lower- and upper-layer neuron generation is changed and some neurons start to co-express makers that are typically restricted to distinct cortical layers (such as *Ctip2* and *Satb2*). These studies provide further evidence that ASCL1 may contribute to glutamatergic neuron specification in normal development, but the regulation and refinement of its downstream transcriptional targets is not well understood.

An intriguing subpopulation of cortical progenitors in the ventricular zone has been shown to co-express ASCL1 and Neurog2. Although this double-positive population was identified over 20 years ago [69], it has only recently been functionally investigated. In the mouse, these cells maintain symmetric Notch signalling with their neighbours, which results in radial growth of the cortex with no folding, and ultimately the to a lissencephalic brain [70]. Ablation of this ASCL1-Neurog2 positive population disrupts Notch signalling, which perturbs the symmetric neurogenic niche and induces cortical folding [70]. How much this mechanism normally contributes to gyrencephalic brain formation in humans is still unclear and it is likely to work in combination with the expansion of outer radial glia cells, which has been shown to amplify the neuronal output in the human brain [71].

### 2.3. ASCL1 in astroglia cells

**Figure 2.**
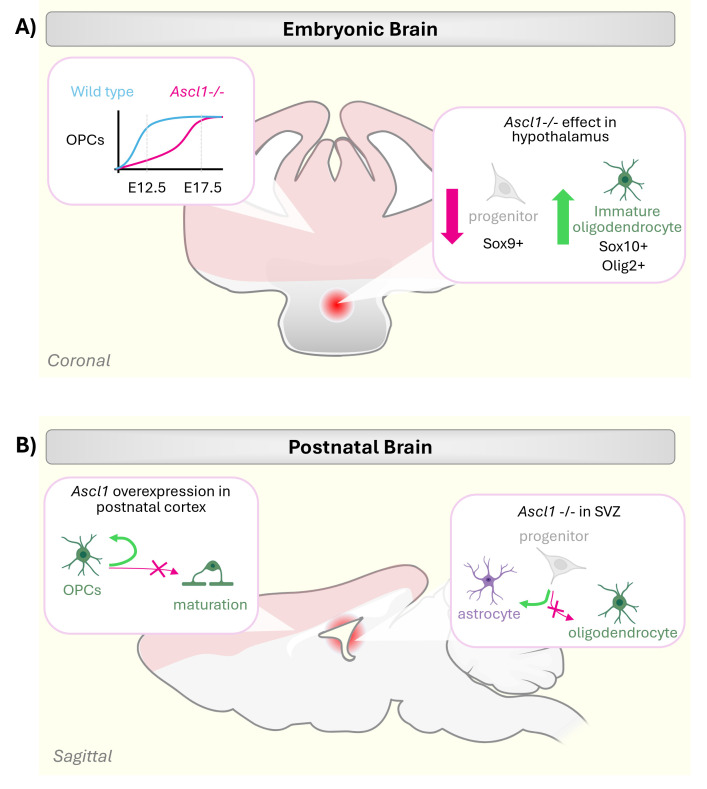
Regional differences in the control of glia by ASCL1. (A) Roles of Ascl1 in glial specification in the embryonic brain. Accumulation of oligodendrocyte precursor cells (OPCs) in the developing telencephalon is delayed, but not abolished, in *Ascl1* knockout mice (*Ascl1−/−*) (left). *Ascl1−/−* mice have fewer Sox9+ progenitors and more Sox10+/Olig2+ differentiating immature oligodendrocytes in the hypothalamus (right). (B) Overexpression of *Ascl1* in the postnatal cortex resulted in increased proliferation of OPCs without a concomitant increase in mature oligodendrocytes (left). Knockout of *Ascl1* (*Ascl1−/−*) in the subventricular zone (SVZ) biases glial progenitors towards astrocytic differentiation, instead of oligodendrocytes (right). Elements created in BioRender (https://BioRender.com/wyl294k).

After the peak of embryonic neurogenesis, which occurs between day 11.5 and 16.5 in the mouse, radial glial cells in the cortex undergo a switch in their output in favour of glia [72]. ASCL1 plays a role in the generation of both oligodendrocytes and astrocytes (figures 1C and 2) [73,74].

ASCL1 is expressed in embryonic and neonatal oligodendrocyte precursor cells (OPCs) *in vitro* [75–77] where it induces expression of several key OPC genes, such as *Sox9* and *Hes5* [78,79]. Overexpression of ASCL1 in neural progenitor cells *in vitro* appears to drive differentiation towards both neurons and oligodendrocytes [58,75]. The heterogeneous response to ASCL1 may be caused by differences in the progenitor state at the point of induction, but the mechanism is not clear. Knockout of ASCL1 in the mouse results in phenotypes that point to contradictory roles. *Ascl1−/−* embryos have fewer OPCs in the telencephalon at day 12.5, although this defect is transient and compensated for by day 17.5 [80] (figure 2A). Conditional knockout in the dorsal SVZ inhibits oligodendrocyte production and biases differentiation towards astrocytes [73] (figure 2B). Overall, these results indicate that ASCL1 is required for differentiation, but not for specification of OPCs [37,58,73,80]. However, other works showed that *Ascl1−/−* mice have fewer Sox9+ cells (astrocyte progenitors) and more Olig2+ and Sox10+ immature oligodendrocytes in the hypothalamus [81] (figure 2A), which would point towards a role in maintaining the OPC population. Thus, in the absence of ASCL1, the progenitors are correctly specified, but they transition out of the proliferative progenitor state too early. It is possible that regional differences might explain this contradiction, but it is not clear how such regional specification of ASCL1 function is achieved. ASCL1 overexpression studies have also resulted in contradictory conclusions about its function. Virus-mediated delivery of ASCL1 into the hippocampus drives oligodendrocyte differentiation from adult progenitor cells [82]. However, in the early postnatal cortex, ASCL1 overexpression resulted in increased proliferation of OPCs without a concomitant increase in mature oligodendrocytes [83] (figure 2B). Notably, this study reported no change in the proliferative status of astrocytes after ASCL1 overexpression, suggesting its effects on the cell cycle is specific to OPCs. It is yet unclear whether ASCL1 behaves differently in OPCs from different brain regions or whether this partially reflects a potential dual role in proliferation and differentiation.

ASCL1 is also implicated in the generation of astrocytes from radial glia progenitors [74]. Lineage tracing in the spinal cord showed no astrocytes descended from ASCL1+ progenitors [45], while clonal fate mapping experiments indicated that ASCL1+ progenitors give rise to both astrocytes and oligodendrocytes [46] (figure 1C). This discrepancy could be caused by differences in the reporter lines used: the earlier study utilized BAC reporter line, while the latter employed the *Ascl1^CreER^* knock-in line. The knock-in line was separately shown to label astrocytes derived from an Ascl1-expressing progenitor in the cerebellum [36], and may better reflect the endogenous expression of Ascl1. Indeed, ASCL1+glial progenitor cells, especially in the dP3–dP5 domains of the developing spinal cord, contribute to dorsal astrocytes in both the white and grey matter [46] (figure 1C). Interestingly, sparse labelling experiments show that while astrocytes in both regions derive from ASCL1+ progenitors, the mature cells display a clonal distribution, suggesting that each progenitor only yields one or other astrocyte subtype [46]. Thus, ASCL1 expression may occur in both cells that are actually divergent products of an earlier bipotent progenitor. This again underlines the wide-ranging and context-dependent roles of this transcription factor.

## 3. ASCL1-mediated differentiation of embryonic and neural stem cells

Embryonic (ESCs) and neural stem cells (NSCs) can recapitulate many developmental processes *in vitro* and allow more detailed genetic and molecular analysis of TF activity than in *in vivo* experiments. ESCs—and, more recently, induced pluripotent stem cells (iPSCs)—have been used as a starting point to study the developmental roles of ASCL1 [84–87]. During ESC-derived neurogenesis, ASCL1 is transiently upregulated in both human and mouse cells [88–92] and it can be further stabilized by Notch inhibition (by γ-secretase inhibitor treatment) [86]. ASCL1 is specifically required for neuronal differentiation of human iPSCs and its deletion causes both an increase in the number of cycling progenitors and a decrease in the number of differentiated neurons [86]. While the onset of endogenous ASCL1 expression in human iPSC (hiPSC) differentiation varies according to the protocol, it typically occurs between 18 and 25 days and always prior to the onset of neurons, which mature weeks later. Forced expression of ASCL1 can accelerate the process and can produce *Tubb3-* and *Map2*-positive neurons within 8−9 days of induction in hiPSCs [93] and within 2−3 days in mouse ESCs [87,92].

Differentiation can be enhanced by co-expression with additional factors, such as Brn2 and Myt1l, which also potentiate ASCL1 function during fibroblast-to-neuron reprograming [93,94]. In human cells, Neurog2 and Neurod1 are the most potent drivers of neuronal differentiation when overexpressed, even though deletion of endogenous *Neurod1* does not inhibit neuronal differentiation [58,95–98]. Interestingly, this is not the case in mouse ESCs, where ASCL1 overexpression results in very efficient neuronal differentiation [87,92,99,100]. Comparison of ASCL1 and Neurog2 activity in mouse ESC-derived embryoid bodies showed that the majority of binding sites are common to both TFs, but distinct transcriptional programs are induced by binding at a minority of specific sites [87,101]. Local chromatin remodelling at these sites facilitates the recruitment of cooperative TFs. ASCL1 activates genes such as *Tfap2b* and *Phox2b* (noradrenergic markers) and *Tlx3* and *Arx* (cortical interneuron markers), and Neurog2 induces genes like *VAChT* and *Olig2* (spinal motor neuron markers) and *Ret* and *Ntrk1* (sensory neuron markers) [87]. How specificity of these similar proneural TFs is achieved is an active question in the field. Substitution of ASCL1 bHLH domain with that of Neurog2 redirected the binding to Neurog2 sites, indicating that intrinsic differences in the DNA binding domain play a major role [101]. The link between intrinsic motif preference and divergent transcriptional regulation was also shown in mESCs and mouse embryonic fibroblast reprogramming [99,102]. These findings are remarkable given the similarity of the consensus motifs recognized by these and other bHLH TFs and they highlight that subtle changes in affinity for different DNA binding sites can have significant effects on cellular phenotype when scaled across the whole genome.

Interestingly, neurons derived from pluripotent cells via expression of ASCL1 alone are mainly excitatory and show minimal expression of GABAergic interneuron markers. However, ASCL1 can induce a diverse range of neuronal phenotypes when coupled with other TFs. ASCL1 co-expression with Dlx2 or with a combination of Lhx6, Dlx5, FoxG1 and Sox2 produces GABAergic interneurons [102–105], while co-expression of ASCL1 and LMX1A yielded mature dopaminergic neurons [85,106]. High throughput screening using a dCas9 activation system in human ESCs identified other factors that potentiate the effects of ASCL1, including LHX6 LHX8, E2F7 and NKX2.2 [107]. Among these, NKX2.2 is of particular interest since its expression and function in embryonic brain development partially overlap with that of ASCL1 in GABAergic neurons specification and OPC development [58,108]. Establishing combinations of TFs for forward programming is essential to refine *in vitro* generation of defined cell types and is an area of active research in both academia and industry.

## 4. Reprogramming of specialized cell types

ASCL1 is a prime candidate for reprogramming strategies aimed at producing neurons both *in vitro* and *in vivo* (tables 1 and 2). Reprogramming can either be via direct conversion, also called transdifferentiation, or via a multipotent intermediate, which then differentiates along a trajectory that is similar to physiological development [9,129]. Transdifferentiation entails direct reprogramming of somatic cells into mature neurons, which completely overrides the original cell identity and may proceed via an artificial intermediate state [10,97,130–132]. Whether transient proliferation occurs also during direct fate conversion and whether a cycling phase plays an active role in reprogramming are still open questions. Moreover, although avoiding proliferative intermediates might seem a safer option from a clinical perspective, this approach requires more starting material to achieve the same neuronal yield for regeneration. ASCL1-mediated reprogramming to neurons has mainly used fibroblasts and glia as the cells of origin, but has also been demonstrated in hepatocytes, blood mononuclear cells, T cells and prostate cancer cells (tables 1 and 2) [10,70,109,110,112,123,124,126,133–136]. Converting cells between developmentally related lineages can minimize the barriers to reprogramming, but transdifferentiation across germ layers is possible and greatly expands the available starting material for therapeutic strategies [137].

**Table 1.** Summary of key neuronal reprogramming experiments in mouse.

	cell of origin	reprogramming factors	cell product	efficiency	ref.
**fibroblasts**	MEFs and postnatal fibroblasts (*in vitro*)	Ascl1, Brn2, Myt1l	excitatory glutamatergic neurons	40−50%	[94]
	MEFs (*in vitro* and *in vivo*)	Ascl1, Lmx1a, Nurr1	dopaminergic neurons	*in vitro:* 18 ± 3% *in vivo:* 40%	[109]
	MEFs (*in vitro* and *in vivo)*	Ascl1, Brn2, Myt1l, Lhx3, Hb9, Isl1, Ngn2	spinal motor neurons	*in vitro:* 5−10%	[110]
	MEFs (*in vitro*)	Ascl1, Myt1l, Isl2, Klf7, Ngn1	nociceptor neurons	14−16%	[111]
	MEFs and adult fibroblasts (*in vitro* and *in vivo*)	Ascl1, Foxg1, Sox2, Dlx5, Lhx6	GABAergic interneurons	*in vitro*: 15−20% *in vivo*: 15−20%	[112]
	MEFs and postnatal fibroblasts (*in vitro)*	Ascl1	excitatory neurons	10−15%	[113]
**astrocytes and glia**	astrocytes (*in vitro*)	Ascl1, Lmx1b, Nurr1	dopaminergic neurons	18.2 ± 1.5%	[8]
	astrocytes (*in vivo*)	Ascl1	excitatory and inhibitory neurons	*in vitro:* 76.8 ± 6.4% *in vivo:* midbrain 93.1 ± 1.7%; striatum 64.4 ± 3.4%; somatosensory cortex 93.9 ± 1.2%	[114]
	striatal NG2 glia (12–16 weeks) (*in vivo*)	Ascl1, Lmx1a, Nurr1	excitatory and inhibitory neurons	46.8% ± 2.9%	[115]
	astrocytes (2–3 months) (*in vivo*)	Ascl1, Nurr1	excitatory neurons	70%	[116]
	midbrain astrocytes (P5−7) (*in vitro* and *in vivo*)	Ascl1(S–A), Phox2b, AP-2α, Gata3, Hand2, Nurr1, Phox2a	noradrenergic neurons	55.3% ± 3.7%	[117]
	spinal cord astrocytes (P2−3) (*in vitro*)	Ascl1, forskolin, dorsomorphin	ventral spinal cord interneurons	55.15%	[118]
	cortical astrocytes (P5) (*in vivo*)	Ascl1(S–A), Bcl2	fast spiking parvalbumin interneurons	80%	[119]
	hippocampal and cortical astrocytes (*in vivo*)	Ascl1, Dlx2	inhibitory neurons	70−80%	[120]
	Müller glia (*in vitro*)	Ascl1	neurogenic retinal progenitors	25−30%	[57]
	Müller glia (P12–P14) (*in vivo*)	Ascl1, retinal injury	neurogenic retinal progenitors	20%	[121]
	Müller glia (4–6 months) (*in vivo*)	Ascl1, retinal injury, HDACi	neurogenic retinal progenitors	50%	[122]
**other**	postnatal and adult hepatocytes (*in vitro*)	Ascl1, Brn2, Myt1l	excitatory neurons	postnatal: 12.5−15% adult: 2.7% ± 1.4%	[123]
	prostate cancer cells (*in vitro*)	Ascl1, Enzalutamide	neuronal stem cell		[124]

**Table 2.** Summary of key neuronal reprogramming experiments in human cells.

	cell of origin	reprogramming factors	cell product	efficiency	ref.
*fibroblasts*	embryonic and postnatal fibroblasts (*in vitro*)	Ascl1, Brn2, Myt1l, NeuroD1	excitatory neurons	2−4%	[93]
	embryonic and postnatal fibroblasts (*in vitro*)	Ascl1, Myt1L, Neurod2, miR-9/9*, miR-124	excitatory and inhibitory neurons	80%	[125]
	embryonic and adult fibroblasts (*in vivo* and *in vitro*)	Ascl1, Lmx1a, Nurr1	dopaminergic neurons	embryonic fibroblasts 10 ± 4–6 ± 2% healthy-PD donors adult fibroblasts: 5 ± 1%–3 ± 1%	[109]
	embryonic fibroblasts (*in vitro* and *in vivo*)	Ascl1, Brn2, Myt1l, Lhx3, Hb9, Isl1, *Ngn2*	spinal motor neurons	0.05%	[110]
	embryonic and postnatal fibroblasts (*in vitro*)	Ascl1, Lmx1a, Brn2, Myt1l, Foxa2	dopaminergic neurons	~10%	[126]
	embryonic and postnatal fibroblasts (*in vitro*)	Ascl1	excitatory neurons	10−15% (co-culture with glia improved morphology and functionality)	[113]
	embryonic fibroblasts (*in vitro*)	Ascl1, Brn2, Myt1l, miR−124	excitatory and inhibitory neurons	80−90%	[127]
	iPSCs and adult fibroblasts (*in vivo* and *in vitro*)	Ascl1, Foxg1, Sox2, Dlx5, Lhx6	telencephalic GABAergic interneurons	iPSCs: 30% adult fibroblasts: 25%	[112]
	adult fibroblasts (*in vitro*)	Ascl1, Myt1l, Isl2, Klf7, Ngn1	nociceptor neurons	control donors: 16.5 ± 1.1 familial dysautonomia patients: 14.1 ± 1.1	[111]
	embryonic and adult fibroblasts (*in vitro*)	Ascl1, Ngn2, Nkx2.2, Fev, Gata2, Lmx1b	serotonergic neurons	58.4 ± 4.2%	[128]
	adult fibroblasts (*in vitro* and *in vivo*)	S-AAscl1, Phox2b, AP−2α, Gata3, Hand2, Nurr1, Phox2a	noradrenergic neurons	14%	[117]

### 4.1. Glia reprogramming

Glial cells are among the most investigated sources to generate neurons both *in vitro* and *in situ*, owing to their shared developmental origins with neurons and their abundance in the brain [138]. Astroglia and striatal NG2 glia cells have been directly converted into mature neurons *in vitro* and *in vivo* by targeted overexpression of ASCL1 [114,115,139]. These induced neurons (iNs) integrated into established neural circuitry, but the reprogramming efficiency varied depending on extrinsic factors such as the age of the animal, the starting cell, the specific targeted brain region and the presence of injury. For example, in the retina, ASCL1 overexpression was no longer sufficient to drive neurogenesis from Müller glia after postnatal day 16 [57,121]. This loss of neurogenic competence with age is likely due to epigenetic factors that limit regeneration by altering the interaction between ASCL1 and its target genes. Indeed, treatment with a histone deacetylase inhibitor (trichostatin A) altered chromatin accessibility in Müller glia cells and facilitated reprogramming towards retinal neurons in adult mice [122]. Transcriptional repressors and epigenetic modifiers, such as the REST complex, may prove to be useful targets when trying to facilitate reprogramming against established epigenetic barriers.

The regional diversity of glia also significantly influences their response to ASCL1, as specific cellular contexts impact efficiency and neuron-subtype acquisition. Astrocytes in the striatum are more amenable to neuronal reprogramming after stroke-like injury than astrocytes in the cerebral cortex, which tend to stall before becoming transit-amplifying cells [140]. Moreover, astrocytes from the cortex and the cerebellum can be reprogrammed to a mix of GABAergic and glutamatergic neurons [138,141–143], whereas spinal cord astrocytes are typically reprogrammed to spinal cord V2 interneuron-like cells [118]. Region-specific differences in the glial cells of origin were also preserved in neuronal reprogramming experiments with NEUROG2 [144]. Combinations of TFs inspired by developmental programmes have been used to generate relevant neuronal subtypes *in vitro* and *in vivo*. For example, astrocytes expressing ASCL1 along with PITX3 or LMX1B and NURR1 can be reprogrammed into midbrain dopaminergic neurons [8,135], while combination of ASCL1 with Dlx2 in reactive glia can generate interneurons that stably integrate into the hippocampal circuit [120]. These two strategies alleviate the symptoms of Parkinson’s disease and mesial temporal lobe epilepsy, respectively, in mouse models [118].

After acute injury, reactive gliosis and inflammation trigger the proliferation and fate plasticity of astrocytes, which, in turn, might favour astrocyte-to-neuron conversion. Indeed, reactive astrocytes show gene expression of neural stem cell-associated genes [145,146], and, when cultured *in vitro*, they give rise to self-renewing multipotent neurospheres [147–149]. Astrocytes in the striatum can produce ASCL1+ transit-amplifying precursor cells in response to injury, similar to neurogenesis from radial glia in the adult SVZ [150]. Injury to the neonatal cerebellum produces an *ASCL1*^+^ transitory state with neurogenic potential from a normally gliogenic *Nestin*-expressing progenitor (NEP) in the Bergmann glia layer [38]. The signalling pathways that regulate this response to injury are yet to be fully characterized.

### 4.2. Fibroblast reprogramming

Direct reprogramming of fibroblasts into neurons via ASCL1 overexpression has been extensively reviewed elsewhere [10,70,109,110,112,126,134,135] (tables 1 and 2). While ASCL1 is not the only TF capable of driving cellular reprogramming, it has been the focus of substantial research in the field. Marius Wernig’s group showed that overexpression of Brn2, ASCL1 and Myt1l (also known as the ‘BAM factors’) was sufficient to reprogram mouse and human fibroblasts into excitatory neurons [93,94,151]. Recent work has shown that induced expression of the endogenous BAM factors using dCas9-VP64 can substitute for exogenous overexpression [152]. Further study revealed that ASCL1 is capable of inducing reprogramming on its own [113], and neither Brn2 nor Myt1l alone achieved any morphological changes in MEFs [151]. Myt1l plays a specific role in suppressing alternative fates, which would otherwise strongly reduce efficiency and fidelity of neuronal reprogramming. Indeed, single cell analysis over a time course of fibroblast-to-neuron conversion showed that the initial transcriptional response to ASCL1 was relatively homogenous, but a divergent myogenic programme was activated by day 20−22. This was counteracted by Myt1l, which suppresses alternative fates and stabilizes the neuronal identity [97,153,154]. The myogenic differentiation could be caused by an incomplete shutdown of the mesodermal fibroblast identity or by a direct role of ASCL1 in activating myogenic genes, which are usually bound by the related myogenic bHLH, MYOD1. Indeed, the ability of Ascl1 to initiate changes in accessibility and transcription at myogenic loci has been employed to enhance the efficiency of cardiac reprogramming in association with MEF2C [155].

Clearly ASCL1 can reprogram a wide range of starting cells into neurons, but there is yet no precise logic to describe what makes one cell more amenable to reprogramming than any other. Although ASCL1 robustly reprograms fibroblasts and astrocytes, keratinocytes appear resistant to conversion [151]. An epigenetic explanation was partly characterized in the initial work in fibroblasts: a unique trivalent chromatin signature, comprising H3K4me1, H3K27ac and H3K9me3, predicts the permissiveness of cells for ASCL1 binding. In cell types with lower levels of this trivalent chromatin state, like the keratinocyte, ASCL1 binding is attenuated, and it cannot initiate neuronal differentiation. However, more work is needed to establish this as a generalizable explanation for ASCL1 activity, as ASCL1 binding in ESCs appears not to show a preference for this trivalent modification and H3K9me3 was not enriched at ASCL1 binding sites [99].

Comparative studies of glia and fibroblasts have not revealed any unifying principles of competence for reprogramming and instead point to cell type-specific mechanisms. For example, although most genomic regions bound by ASCL1 in astrocytes and MEFs are shared, the dependence on co-factors is cell type specific. The Zinc finger TF, Zfp238, is a key downstream target of ASCL1 in fibroblasts and can partially substitute for ASCL1 during direct reprogramming of fibroblasts into neurons, but not in astrocytes [131,151]. Instead, ASCL1-mediated conversion of astrocytes required its downstream target Neurod4, which was unable to elicit neuronal conversion in MEFs, unless coupled with Insm1 [156].

## 5. ASCL1 in brain tumours and paediatric neuroblastomas

ASCL1 expression reaches its peak during development, and it is downregulated in the adult, with exception of some neurogenic niches. Re-expression of ASCL1 has been observed in several brain tumours [157–159], notably in a subset of gliomas and glioblastomas [160–163], implying either a direct developmental origin of the cancer cells or, more likely, a recapitulation of embryonic pathways [164–166]. The dual role in proliferation and differentiation that ASCL1 plays in development has been shown in tumours too, making ASCL1 both an oncogene and a tumour suppressor. Loss of ASCL1 in glioblastoma stem cells reduces their proliferation *in vitro* [167] and, to a lesser extent, *in vivo* in mice, where Ascl1 works redundantly with the bHLH TF Olig2 to sustain tumour growth [168,169]. On the other hand, high ASCL1 expression promotes differentiation of glioblastoma stem cells into neurons [161,163] and promotes tumour cell migration *in vivo* [168]. Endogenous levels of ASCL1 in glioma and glioblastoma are variable and tend to be higher in tumours of the ‘proneural’ class, characterized by neural progenitor cell (NPC) features. By contrast, mesenchymal subtype tumours, which are enriched for astrocyte and injury-like signatures, generally lack ASCL1 [161,162]. Proneural glioblastomas are more responsive to differentiation with Notch and Wnt inhibitors [161,170,171], indicating that the interplay between these developmental pathways is key for tumour progression and self-renewal. Importantly, forced ASCL1 in the mesenchymal subtype does not drive neuronal differentiation, but instead induces a neuroendocrine phenotype coupled with increased malignancy [162], which has important clinical implications for developing safe and effective interventions for glioblastoma.

Neuroblastoma is a paediatric tumour of the peripheral nervous system, originating most frequently in the adrenal glands or other organs along the spine. Like glioblastomas, neuroblastoma can be characterized as either adrenergic (neuronal) or mesenchymal, reflecting the multipotency of the neural crest lineage from which these tumours originate [172]. Neuroblastoma cell identity is sustained by a set of TFs that cross-regulate each other to establish a core regulatory circuitry (CRC). In adrenergic neuroblastoma cells, ASCL1 is part of the CRC that maintains cell identity [173,174] and controls cell proliferation [175]. Together with the other CRC genes, like *Phox2b*, *Hand2* and *Gata3*, ASCL1 associates with enhancers that sustain the expression of these genes through a positive regulatory feedback loop. In ASCL1 knockout cells, CRC TFs show altered expression levels and reduced chromatin association [175–177], highlighting that the crosstalk between these factors occurs at multiple levels. Interestingly, the oncogene *MYCN* is also part of the CRC, and neuroblastomas with *MYCN* amplification and high ASCL1 levels exhibit the worst prognosis [174]. How ASCL1 cooperates with oncogenic mutations is still unclear: ASCL1 has a direct effect on chromatin remodelling and gene expression [161] and whether the presence of mutations in coding and non-coding regions of the genome can impact ASCL1 activity or co-opt it into the tumourigenic circuit is still to be explored.

## 6. Molecular mechanisms of ASCL1 activity and regulation

### 6.1. ASCL1 as a pioneer factor

**Figure 3.**
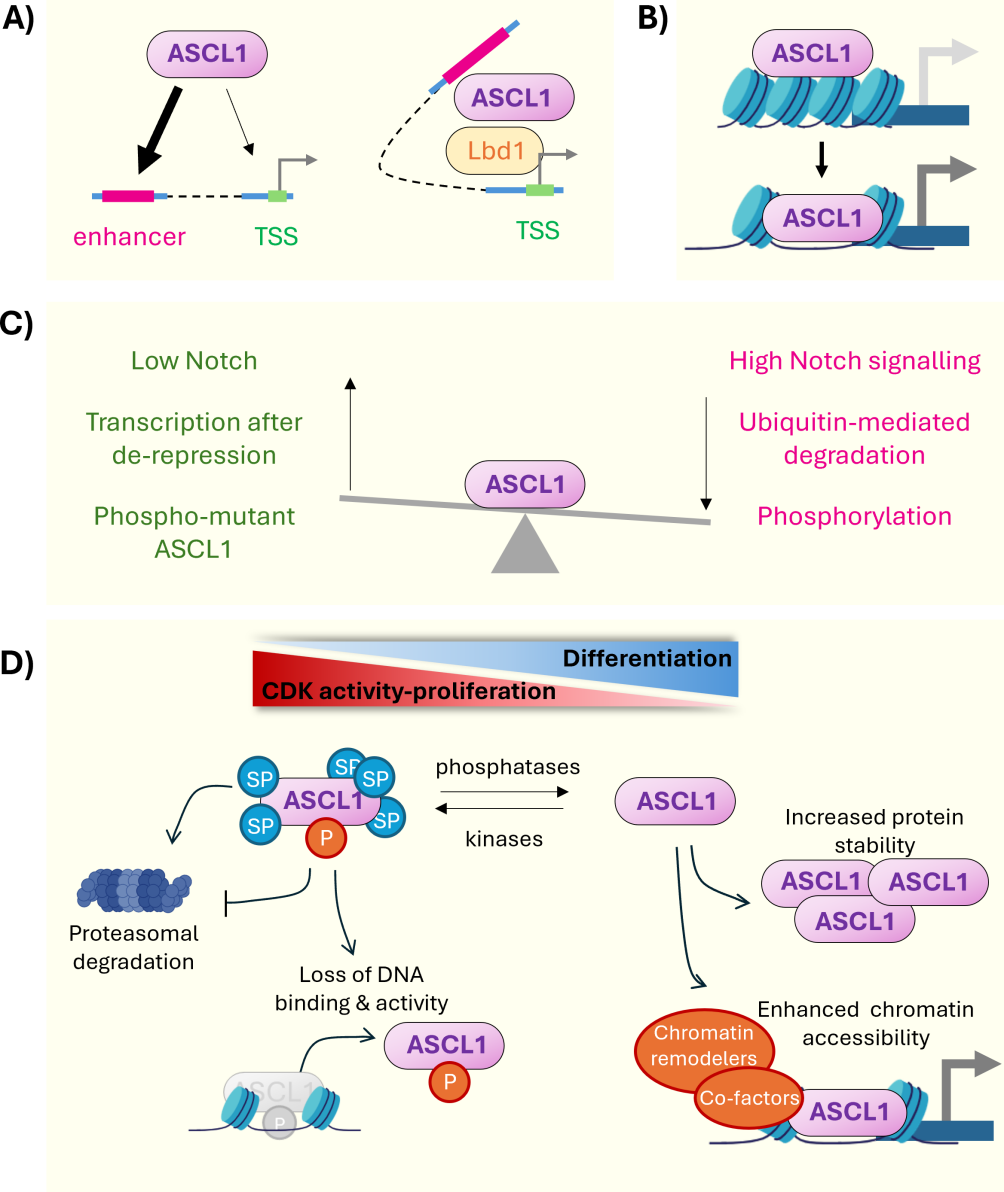
Models of ASCL1 activity and regulation. (A) ASCL1 binding sites are biased towards enhancers rather than transcription start sites (TSS). As a result, ASCL1 recruits other factors, such as Lbd1 to promote long distance TSS recruitment and gene transcription. (B) ASCL1 can bind nucleosome-dense chromatin that would be inaccessible to other TFs. Binding of ASCL1 to these sites has been shown to precede chromatin opening, and eventual gene expression. (C) ASCL1 is regulated by several interacting mechanisms. Activating stimuli (in green) include low notch signalling and de-phosphorylation of ASCL1. Repressive control mechanisms (in magenta) include high Notch signalling, phosphorylation and ubiquitin-mediated degradation. (D) Schematic representation of how phosphorylation of ASCL1 integrates multiple regulatory mechanisms, including proteasomal turnover, DNA binding and chromatin accessibility. Elements created in BioRender (https://BioRender.com/zrjm9ss).

Much work has been done to understand how ASCL1 functions in development, differentiation and disease. TF binding analysis in cell types such as neural stem cells and neural-committed neuroblastoma cells indicate that ASCL1 binds predominantly at enhancer and intergenic regions, rather than promoters. This suggests that ASCL1 likely interacts with the basal transcriptional machinery through long-range interactions [177,178]. These binding events bring distant loci into closer contact via three-dimensional (3D) chromatin looping, and likely require interactor proteins, as described for ASCL1–Lbd1 interaction in the developing pituitary gland [179] (figure 3A).

A key question which has motivated many recent research contributions is the extent to which ASCL1 acts as a ‘pioneer’ TF—that is, a TF that can bind inaccessible chromatin and drive changes that facilitate the activity of downstream effectors in neural differentiation (figure 3B). Integration of ASCL1 binding data with chromatin accessibility and nucleosome footprint data revealed that ASCL1 binds sites associated with closed chromatin at the onset of reprogramming, which then become more accessible as cells progress towards a neuronal identity [151,180]. Taken with the observations that ASCL1 alone can reprogram MEFs into neurons, ASCL1 clearly meets the definition of a pioneer factor [181]. The more interesting question arises of why ASCL1, and pioneer factors more generally, are only capable of driving reprogramming in some cell types and not others, despite their ability to induce major chromatin reconfigurations?

Part of the answer may lie in the cell type specific availability of chromatin modifiers necessary for ASCL1 to remodel the chromatin. ASCL1, like other pioneer factors, does not have any intrinsic enzymatic activity required for remodelling chromatin and relies on the recruitment of other co-factors. The SWI/SNF complex is a likely candidate in neural progenitors, as its subunit composition is associated with changes in differentiation [182]. However SWI/SNF and ASCL1 co-bind mainly at regions of the DNA that are already partially permissive [86,183]. Histone acetyl-transferase enzymes have also been found associated with ASCL1 in MEFs reprograming. Association with Tip60, for example, results in acetylation of H2A.Z on a specific subset of genes that are off in MEFs but exhibit a bivalent signature, enriched for both H3K4me3 and H3K27me3 [184]. However, whether ASCL1 association with acetylation enzymes specifically contributes to its pioneer activity is still unclear.

Another potential explanation for the selective ASCL1 ability to reprogram certain cell types and not others, despite its pioneer activity, might lie in the cell type specific sequences recognized on closed or partially accessible chromatin. The consensus motif for bHLH TFs is the E-box, CANNTG and different bHLH TFs display preference for certain combinations of the central two nucleotides [185,186]. Work in ESCs showed that different versions of this motif are bound by ASCL1 in open versus closed chromatin, with the CAGGTG motif being enriched on closed chromatin and CAGCTG being enriched on nucleosome-free regions [99,101]. However, in fibroblasts, CAGCTG appears more frequently in ASCL1 binding sites in closed chromatin, indicating cell-type specific differences [151]. Moreover, Ascl1 peaks associated with closed chromatin in ESCs contained multiple E-boxes (3–4 times more frequently than in open regions), which may enhance the binding. How pioneer factors recognize specific regions on closed chromatin is still a matter of debate, but insights from structural biology have been reviewed recently [187,188]. For some bHLH proteins, co-factor binding is required to stabilize assembly at nucleosome-bound DNA, as seen for c-Myc. Indeed ASCL1 displays greater affinity for nucleosomes in complex with Tcf3 [189]. In addition, bHLH proteins appear to prefer degenerate motifs at nucleosome-occupied sites [190]. The relative affinity for nucleosome-occluded DNA also relates to the extent of folding of the basic domain as the TF-DNA complex forms. ASCL1 has a shorter basic-helix domain than other bHLHs, and this facilitates lower energy binding at nucleosomal DNA [190]. However, the related pioneer bHLH TF, MyoD, has a long basic helix1 region, suggesting that helix length alone is not predictive of pioneer activity. Further studies to this end are needed.

### 6.2. Regulation of ASCL1 expression and activity

To ensure temporal and spatial control of neurogenesis during brain development, the expression and activity of ASCL1 must be tightly regulated (figure 3C). ASCL1 expression oscillates in antiphase with Notch signalling effectors and it does so by activating the expression of Dll ligands that, in turn, will activate the Notch receptor on adjacent cells. Notch activation releases the Notch intracellular domain (NICD) to upregulate transcription of *Hes1*, *Hes5* and *Hes6*, which inhibit proneural bHLHs. Via this cycle of lateral inhibition, ASCL1 expression in neural progenitors oscillates several times across the cell cycle following a period of 2−3 h. This dynamic equilibrium of activation and inhibition retains progenitors in an undifferentiated state, and differentiation is only initiated once ASCL1 levels are elevated and stabilized [78,191–193]. Hence, ASCL1 over-expression often results in progenitor cell cycle exit and differentiation [194]. However, ASCL1 can also have a pro-proliferative effect [78]; several transcriptional targets of ASCL1 in neural stem cells are genes that contribute to the proliferation of progenitor cells, such as *E2f1, Cdk1* and *Cdk2* [4].

Levels of ASCL1 are modulated both by transcriptional repression and increased ubiquitin-mediated degradation (figure 3C,D) [195]. The short half-life of ASCL1 protein is necessary for oscillatory control and allows manipulation of neuroblast fate decisions by targeting ASCL1 protein degradation. Stabilization of ASCL1 levels via treatment with Notch inhibitor, DAPT, has been shown to promote neurogenesis both in human neural stem cells and in cancer cells [86,196]. Also, ASCL1 degradation by the E3 ubiquitin ligase, Huwe1, is essential for long-term maintenance of the dormant stem cell pool in the adult brain [197]. Huwe1 knockout causes ASCL1 accumulation, increased Cyclin D1 and re-activation of adult stem cells to a proliferative state. This ultimately leads to long-term depletion of the stem cell pool and impairment of hippocampal neurogenesis in adult mice. Similarly, the HLH protein, ID4 (related to the bHLH proteins, but lacking a basic DNA-binding domain) promotes ASCL1 degradation by sequestering its dimerization partner, Tcf3, leaving ASCL1 more susceptible to degradation by the proteasomal machinery [198]. ASCL1 can be protected from Huwe1-mediated degradation when bound to the chromatin, but not when displaced to the cytoplasm, so subcellular localization of ASCL1 is also important in regulation of its expression and activity [199].

### 6.3. ASCL1 phosphorylation

The levels of ASCL1 protein and its proneural activity are also regulated by phosphorylation (figure 3D). Phosphorylation on serine-proline (SP) sites can prime ASCL1 for degradation [92], likely via ligase recruitment by the phospho-serine residues, a mechanism also observed in other proneural bHLHs, such as Neurog2 and Neurog3 [176,200,201]. We and others have shown that preventing ASCL1 phosphorylation on SP sites, by mutating the serine residues to alanine, promoted neuronal differentiation [202], increased efficiency of fibroblast-to-neuron [160], and astrocyte-to-neuron reprograming [119,203], and enhanced differentiation of both neuroblastoma and glioblastoma cells [163,176].

Because un-phosphorylated ASCL1 is more stable than wild type ASCL1, increased ASCL1 activity upon dephosphorylation have been generally attributed to the accumulation of ASCL1 protein. Intriguingly, however, our recent work showed that the enhanced neurogenic activity of de-phosphorylated ASCL1 is independent of increased protein stability and levels [92]. We found that the primary effect of ASCL1 dephosphorylation during *in vitro* neurogenesis is to facilitate chromatin remodelling and chromatin opening at neuronal sites. Our data also revealed little to no differences in the preferred E-box sequences recognized by wild type and phospho-mutant ASCL1 [92,177]. This indicates that both forms of Ascl1 can potentially bind to shared sites, which are then differentially engaged by phospho-mutant ASCL1, either because of increased strength of binding and/or because of better chromatin remodelling. Hence, we propose a model whereby the phosphorylation status of SP sites influences the electrostatic interactions between ASCL1 molecules and/or between ASCL1 and the DNA, affecting the ability to scan the nucleosome in closed or partially permissive chromatin. Phosphorylation at sites beyond the SP motif also affects ASCL1 expression and activity. Phosphorylation at Ser152 (labelled Ser155 in NP_004307.2) increases ASCL1 stability by promoting association with Tcf3 [204], but it is also predicted to inhibit DNA binding and bHLH activity [5,205] (figure 3D). Thus, integration of multiple phosphorylation sites ensures multilayered and nuanced regulation of ASCL1.

Regulation of ASCL1 by phosphorylation has important implications for understanding its role in cancer, in which cells often have a hyperactive kinase environment [206]. Phosphorylation via cyclin-dependent kinases (CDKs) and other kinases can phosphorylate ASCL1 in both neuroblastoma [176] and glioblastoma stem cells [163]. ASCL1 phosphorylation by ERK leads to glial cell proliferation and astrocytoma, while intermediate levels of ERK activity promotes a ganglioneuroma phenotype [207]. Targeting ASCL1 phosphorylation may present an attractive therapeutic target, but the effect of blocking phosphorylation remains only partially characterized. In glioblastoma, for example, phospho-mutant ASCL1 could not produce fully mature neurons, in part because of the upregulation of ID proteins that can negatively feedback on ASCL1 activity [163]. Thus, additional adjustments might be needed to reach terminally differentiated post-mitotic neurons.

## 7. Conclusion

Since the first identification of the *achaete-scute* mutants in *Drosophila* [208], proneural proteins have attracted considerable attention for their role in nervous system development and in cell fate reprogramming. The mammalian homologue, ASCL1, has taught us much about the intricacies of stem and progenitor cell regulation in the specification of nervous tissue. Cell fate decisions are delicately balanced to ensure proper development of the CNS and PNS, and homeostasis of the adult brain. ASCL1 lies at the heart of these regulatory networks in different cell types, including neural stem cells, GABAergic interneurons, sympathetic adrenergic precursors and glia cells. Emerging research in cell differentiation and cell fate reprogramming, alongside advancement in molecular technologies to study ASCL1 functions, have revealed that ASCL1 activity goes beyond regulation of gene expression at transcriptional start sites. Rather, ASCL1 can establish long-range interactions, participate in 3D genome reorganization and act as a pioneer factor on closed or partially permissive chromatin to initiate a cascade of events leading to cell fate changes. Through chromatin remodelling, ASCL1 can act on different cellular contexts and often exhibits lineage promiscuity in the type of genetic program that it activates. ASCL1 promiscuous binding to non-neuronal loci can be used to enhance reprogramming of non-neuronal cell types, especially when expressed in association with non-neuronal lineage-specific transcription factors [155]. Moreover, further understanding of ASCL1 role in balancing cell fate decisions in different cellular contexts will be essential also to establish ASCL1 pathogenic role in neurological cancers, and in other non-neural cancers, such as lung and prostate cancer [42,124,163,175,176]. In these cancers, ASCL1 expression is often associated with aggressive tumour phenotypes, making it a potential therapeutic target.

Overall, future research should focus on understanding how ASCL1 response is shaped by the cellular contexts and how much contribution comes from chromatin pre-patterning and/or from the presence/absence of specific co-factors. These studies should go alongside an understanding of how different bHLHs achieve their specificity and ensure lineage fidelity in development and in cell fate reprogramming experiments. By addressing these outstanding questions, we can better harness the potential of ASCL1 for therapeutic applications in neurological disorders, regeneration and cancer.

## Data Availability

This article has no additional data.
